# Contact-Free Palm-Vein Recognition Based on Local Invariant Features

**DOI:** 10.1371/journal.pone.0097548

**Published:** 2014-05-27

**Authors:** Wenxiong Kang, Yang Liu, Qiuxia Wu, Xishun Yue

**Affiliations:** 1 College of Automation Science and Engineering, South China University of Technology, Guangzhou, China; 2 Guangzhou Institute of Modern Industrial Technology, South China University of Technology, Guangzhou, China; Plymouth University, United Kingdom

## Abstract

Contact-free palm-vein recognition is one of the most challenging and promising areas in hand biometrics. In view of the existing problems in contact-free palm-vein imaging, including projection transformation, uneven illumination and difficulty in extracting exact ROIs, this paper presents a novel recognition approach for contact-free palm-vein recognition that performs feature extraction and matching on all vein textures distributed over the palm surface, including finger veins and palm veins, to minimize the loss of feature information. First, a hierarchical enhancement algorithm, which combines a DOG filter and histogram equalization, is adopted to alleviate uneven illumination and to highlight vein textures. Second, RootSIFT, a more stable local invariant feature extraction method in comparison to SIFT, is adopted to overcome the projection transformation in contact-free mode. Subsequently, a novel hierarchical mismatching removal algorithm based on neighborhood searching and LBP histograms is adopted to improve the accuracy of feature matching. Finally, we rigorously evaluated the proposed approach using two different databases and obtained 0.996% and 3.112% Equal Error Rates (EERs), respectively, which demonstrate the effectiveness of the proposed approach.

## Introduction

Biometrics identification is a personal identification technology that takes full advantage of inherent physiological or behavioral characteristics of humans compared with traditional authentication, such as passwords or encrypted codes. It exhibits greater security and reliability because biometric features are difficult to duplicate and forge. Moreover, the unique, portable and inherent properties of biometric features have attracted significant attention from many researchers in recent years. Among the possibilities, veins show more distinctive features and merits compared with other biometric features, such as fingerprints, iris and face, as described below.

- Live body identification: vein patterns can only be identified on a live body;
- Anti-interference: veins are an internal feature of the vessel structure under the skin and are thus more tolerant to breakage, pollution and scarring;
- Simple acceptability and anti-counterfeit: because the image acquisition does not require contact during registering and authentication, it induces no health issues and reduces the risk of replication.

Due to the convenience of image acquisition, several vein features in the hand have been well studied, such as finger veins, hand veins and hand-dorsal veins. In particular, the palm veins have gained more attention from researchers due to their more abundant texture information and easy acquisition. Recently, studies of palm veins have focused on feature extraction methods that acquire the salient features more efficiently. The related approaches can be broadly categorized into three groups as follows.

- Geometry-based methods: these methods typically use vascular structure information. Curve-like or line-like features in the vascular structure are generally extracted using spatial methods, such as multi-scale Gaussian matched filtering and scale production [1], maximum curvature points [2], principle curvature [3], mean curvature [4], repeated line tracking [5], EDGF [6], Gabor filter [7]–[10] and vectorgrams of maximal intra-neighbor difference [11]. The vascular radius, length and degree and minutiae coordinates are also extracted. Finally, these features are matched by using the matched pixel ratio [4], the Phase-Only-Correlation [9], [12] or based on some distance measures, for example, the Hamming distance [1], [8], [10] or the Hausdorff distance [13], [14]. However, the presence of thin and blurred lines in the vein image renders accurate extraction and binarization more difficult and may influence the final matching result. In addition, these methods are not invariant to rotation, scaling, or translation of the vein images.
- Statistical-based methods: these methods typically use various statistical data from vein images, such as the image invariant moment [15], [16], the LBP [17] and its variant, for example, the local derivative pattern (LDP) [18], [19], local ternary pattern (LTP) [20], partition local binary pattern (PLBP) [21] and local line binary pattern [22]. These statistical methods can depict a gray histogram distribution of the vein image, but they lose positional information on the vein texture. Thus, block-based strategies are adopted to compensate. However, the use of block-based strategies makes these methods sensitive to changes in translation, rotation and scale, which renders this method unsuitable for contact-free vein recognition.
- Local-invariant-feature-based methods: these methods extract stable local invariant features for matching. Compared with the aforementioned methods, these methods are invariant to rotation,scale and translation, which makes them appropriate for contact-free vein recognition. Pierre *et al.* [23] used SIFT [24] for feature extraction and matching after preprocessing and binarization on vein images. However, because the binarization will result in the loss of useful feature information, some researchers [25]–[27] have directly extracted SIFT and SURF [28] features from the vein image after noise removal and illumination normalization, thereby improving the correct matching rate. Pan and Kang [29] analyzed and compared the performance of three local invariant feature descriptors on the NIR sub-database of the PolyU multispectral palmprint Database [30] and demonstrated that all descriptors showed good performance on contact vein recognition. The SURF algorithm showed the best synthetic performance while ASIFT [31] showed the highest accuracy, which are consistent with theoretical analyses.

**Figure 1:**
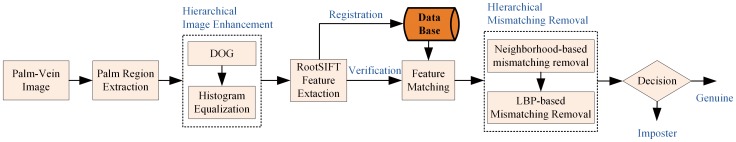
Flowchart of the proposed algorithm.

Reviews of prior research on palm-vein recognition suggest that local invariant features are more appropriate for contact-free vein recognition due to their invariant to changes in rotation, scale and translation. However, many problems remain to be overcome prior to practical application. (1) The extracted feature points are relatively few: due to the blurred and low-contrast texture, the feature points extracted from vein images are relatively few. (2) Many mismatching points can arise: there are many similar structures in vein images, which increases the difficulty of correct matching. (3) Many studies [1], [23], [31]–[34] have extracted a region of interest (ROI) prior to feature point extraction, which does not take full advantage of the local-invariant-feature-based method and causes unnecessary time consumption and the loss of useful feature data. Some useful vein information exists in the finger region and outside the palm region, which is typically excluded from the ROI in traditional methods. To solve the above problems, we have taken the entire palm region as the object of interest and propose a new contact-free vein recognition strategy. The flowchart of our proposed strategy is illustrated in Figure 1.

The key contributions from this paper can be summarized as follows. (1) We propose a new hierarchical vein image enhancement method: Difference of Gaussian-Histogram Equalization (DoG-HE). This method effectively improves the clarity and contrast of texture features in the contact-free vein image, increasing the number of extracted invariant feature points. (2) Our method not only takes full advantage of all the palm-vein information, including finger regions and outside-palm regions, but also increases the freedom of hand layout. Furthermore, the recognition performance is improved by importing RootSIFT [36], which is more robust to changes in scale, translation and rotation. (3) We propose a hierarchical mismatching removal algorithm that further improves the accuracy of feature matching.

This paper is organized as follows. Section 1 presents the details on the preprocessing steps that enhance palm vein recognition using the DoG-HE algorithm after segmenting the entire palm. Section 2 describes the RootSIFT feature extraction and matching approach for contact-free palm-vein identification. Section 3 introduces the proposed hierarchical mismatching removal algorithm, and experimental results and analysis are presented in Sections 4–9. Finally, we summarize this paper and conclude future work in Conclusions.

## Methods

### 1. Preprocessing

The palm-vein images employed in our research were acquired under near-infrared (NIR) illumination; the images generally appear darker with low contrast, and the illumination is not uniform. If the local invariant features are extracted from these images directly, it is difficult to obtain sufficient feature points. Furthermore, the impurities in the background also influence feature extraction, feature matching and the final recognition result. To address these issues, we propose a new preprocessing method that extracts the entire palm region and enhances the vein texture.

#### 1.1 Palm region extraction

For extraction of the palm region, the fingertips and finger valleys are used as landmarks, as in the majority of previous reports, to locate and extract the maximum inscribed rectangular region of the palm, which is taken as the ROI for feature extraction and matching. This approach presents two issues. (1) Palm layout positions are restricted due to the need to locate the fingertips and finger valleys. For example, the fingers must be completely open and the fingers must not touch the boundary to prevent unpredictable errors during the extraction process. (2) Useful feature information can be lost by taking partial rectangular regions of the palm as the ROI. To address these issues, we have taken the entire palm region including the five fingers but excluding the palm region near the wrist for feature extraction and matching.

**Figure 2:**
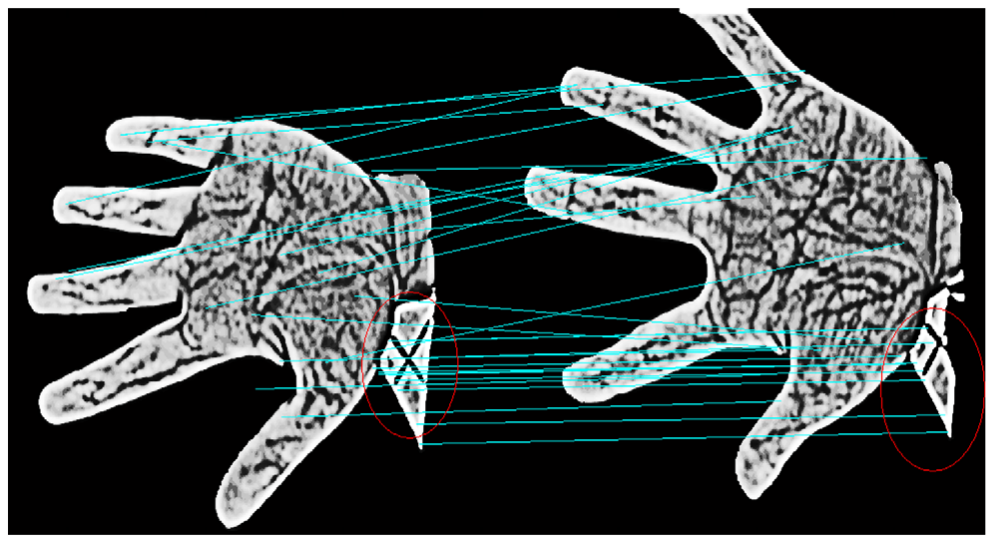
Feature matching before cropping the palm region near the wrist.

**Figure 3:**
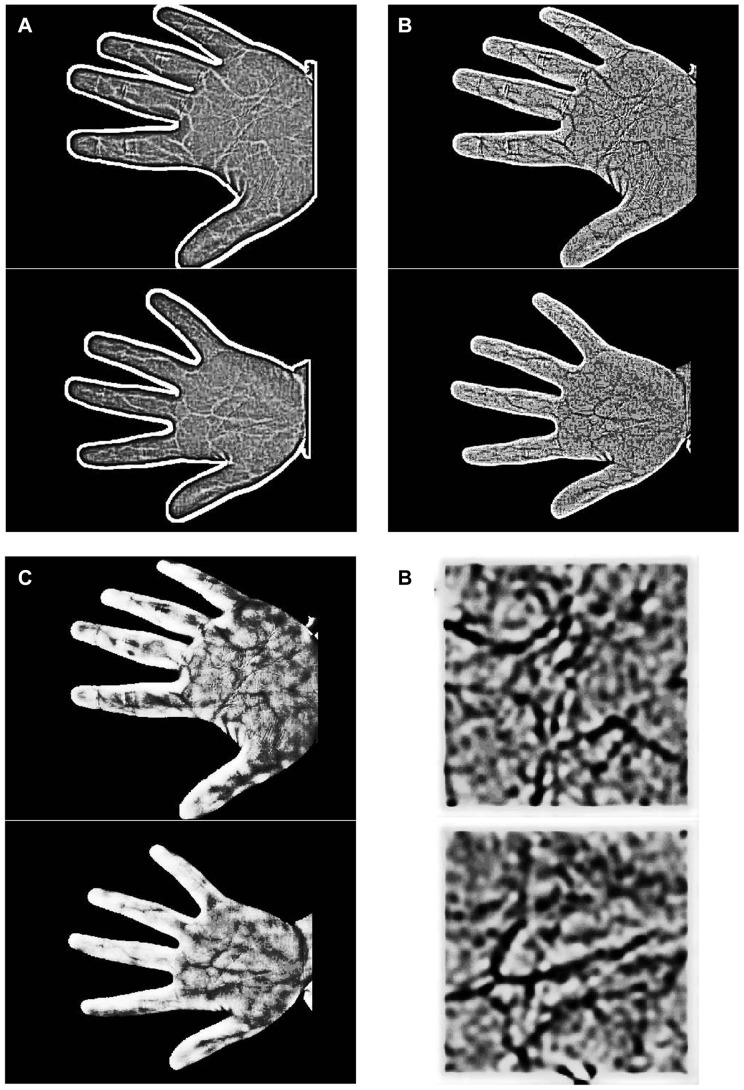
Image preprocessing. (a) Original images; (b) Cropping the palm region near the wrist; (c) DoG filtering; (d) Histogram equalization.

Global threshold methods, such as fixed threshold, mean threshold and OTSU methods, have typically been adopted for palm extraction because they are simple and fast [37]. Among these methods, the OTSU can perform effective segmentation by seeking the optimal threshold from the gray histogram and can segment images with uneven illumination. Thus, we adopted this method to segment the palm region for our research. Following palm segmentation by OTSU, false textures appear near the wrist that are similar to the vein texture and that result in side effects when extracting and matching the local invariant feature points. In Figure 2, we present results for feature point extraction and matching from two vein images segmented by OTSU before cropping the palm region near the wrist. The figure shows many mismatching points near the wrist. Thus, we cropped this region using the following strategy. First, the centroid of the segmented palm region is computed. Second, a region of greater distance from the centroid is cropped. The valid palm region after cropping the region near the wrist is illustrated in Figure 3(b).

#### 1.2 Hierarchical enhancement

***1.2.1 Difference of Gaussian.*** The acquired palm-vein images generally appear blurry and are of low contrast. Therefore, image enhancement is essential before feature extraction. Inspired by Lowe's work [24], in which Gaussian pyramids were constructed and Difference-of-Gaussian (DoG) filters were used to locate key points, we used the DoG filter for vein-image enhancement. The DoG filter is an image enhancement algorithm that involves the subtraction of one blurred version of an original image from another, less blurred version of the original. The two blurred images are obtained by convolving the original grayscale images with Gaussian kernels of two different standard deviations. The DoG filter is defined in two dimensions as follows: 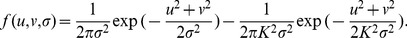
(1)


The majority of sharpening filters are achieved by enhancing the high-frequency signal. However, because random noise is also of high spatial frequency, it will be enhanced together with the image. Blurring an image using a Gaussian kernel suppresses only high-frequency spatial information. The process of subtracting one image from another preserves spatial information that lies between the ranges of frequencies preserved in the two blurred images. Thus, the DoG filter is a band-pass filter that discards all but a few spatial frequencies that are present in the original grayscale image. When the DoG filter is utilized for image enhancement, the radius ratio of the two different Gaussian kernels is typically 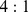 or 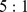. A ratio of 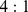 is adopted for our research. Palm-vein images processed by the DoG filter are illustrated in Figure 3(c), which shows that the DoG filter enhances detail but that two further problems still exist in the enhanced image (low contrast and blur), which thus require further processing.

**Figure 4:**
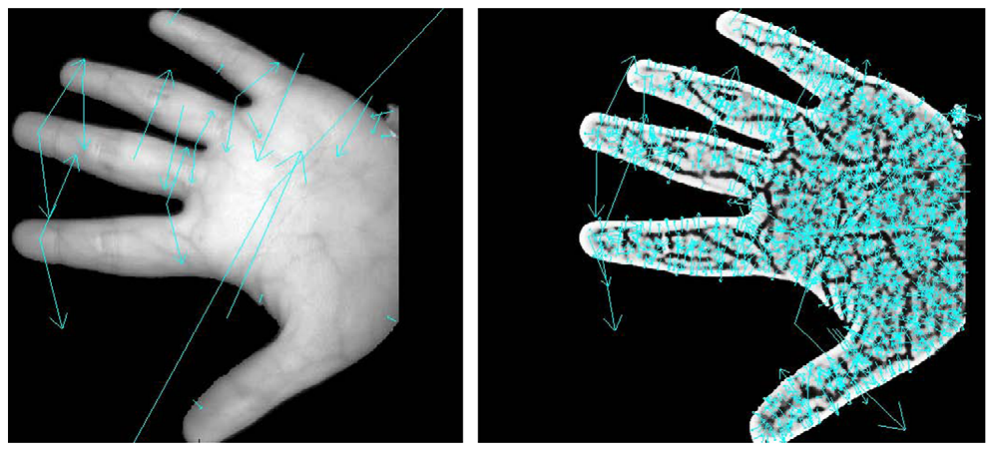
Feature extraction before preprocessing (Left) and after preprocessing (Right).

***1.2.2 Histogram equalization.*** Histogram equalization is a histogram correction method based on a transformation of the cumulative distribution function, which is generally adopted to increase global contrast, in particular where the distribution of gray levels in the image is excessively concentrated to a narrow interval. For example, in Figure 3(c), the distribution of gray levels within the palm region is excessively concentrated and the contrast is very low between the veins and the background, which makes feature extraction more difficult. We thus used histogram equalization to increase the contrast and to highlight the veins. The processed image is shown in Figure 3(d). This figure shows that histogram equalization increases the contrast of the palm-vein region and results in more vein-texture detail, which will enable the extraction of more feature points. Figure 4 presents the resulting differences in feature extraction before and after processing by histogram equalization. Obviously, the number of extracted feature points increases greatly when using our proposed enhancement algorithm.

### 2. Feature extraction and matching

Several changes occur in scale, rotation, translation and illumination in contact-free palm-vein images, in contrast to palm-vein images acquired by a contact acquisition device. Thus, the feature extraction method should be more stable and robust to enable contact-free vein-image recognition. Recently, local-invariant-feature-based methods have been adopted to deal with these issues, for example, the RootSIFT [36] algorithm, a newly proposed local invariant feature algorithm that is very robust to changes in scale, rotation and viewing angle. We adopt the RootSIFT algorithm here for feature extraction and matching in contact-free palm-vein images. RootSIFT [36] and SIFT [24] adopt the same strategy for feature detection and description and include the following steps: (1) scale-space extrema detection; (2) key-point localization; (3) orientation assignment; (4) generation of key-point descriptors; and (5) the formation of several 128-dimension descriptors to represent image features. However, RootSIFT adopts the Hellinger kernel for similarity measurements rather than the Euclidean distance, which brings dramatic improvement in performance [36]. Next, we will analyze the relationship between the Hellinger kernel and the Euclidean distance.

The Hellinger kernel for two 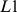 normalized vectors, x and y (*i.e.*, 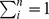 and 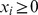), is defined as: 
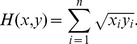
(2)


To maintain invariant illumination, the feature descriptor has been normalized to a Euclidean unit vector. Thus, the relationship between the Euclidean distance and the kernel is defined as follows: 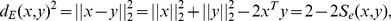
(3)where 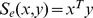. A similarity measure of the two feature vectors, can be implemented by the following two algebraic operations: (1) normalize the SIFT vector to 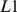 (it was originally unitized to 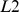 norm); (2) find the square root of each element in the normalized SIFT vector. 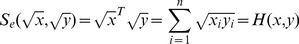
(4)

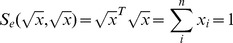
(5)


Therefore, by using the Hellinger kernel, the Euclidean distance can be described as follows: 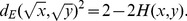
(6)


Thus, the feature vectors' Euclidean distance is mapped to the Hellinger kernel. RootSIFT's feature vectors are calculated by the Hellinger kernel, and the match between two feature points is then judged by the ratio of the closest and the second-closest distance of feature points.

**Figure 5:**
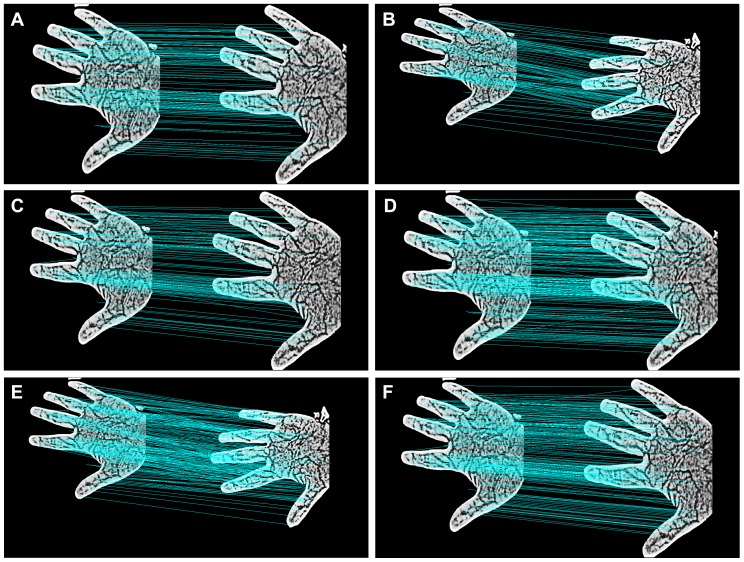
SIFT ((a), (b), (c)) and RootSIFT ((d), (e), (f)) are compared for their robustness against rotation and scale transformations.

The recognition performance is improved by replacing the Euclidean distance with the Hellinger kernel, which has been demonstrated previously [36]. We also performed three matching experiments to evaluate the performance of SIFT and RootSIFT in contact-free vein recognition: (1) two different samples from the same individual without transformation; (2) two different samples from the same individual in which one sample was rotated prior to matching; (3) two different samples from the same individual in which one sample underwent scale transformation prior to matching. As shown in Figure 5, we obtain more optimal matching when using the RootSIFT method, which also demonstrates that RootSIFT is more robust to rotation and scale transformations than SIFT.

**Figure 6:**
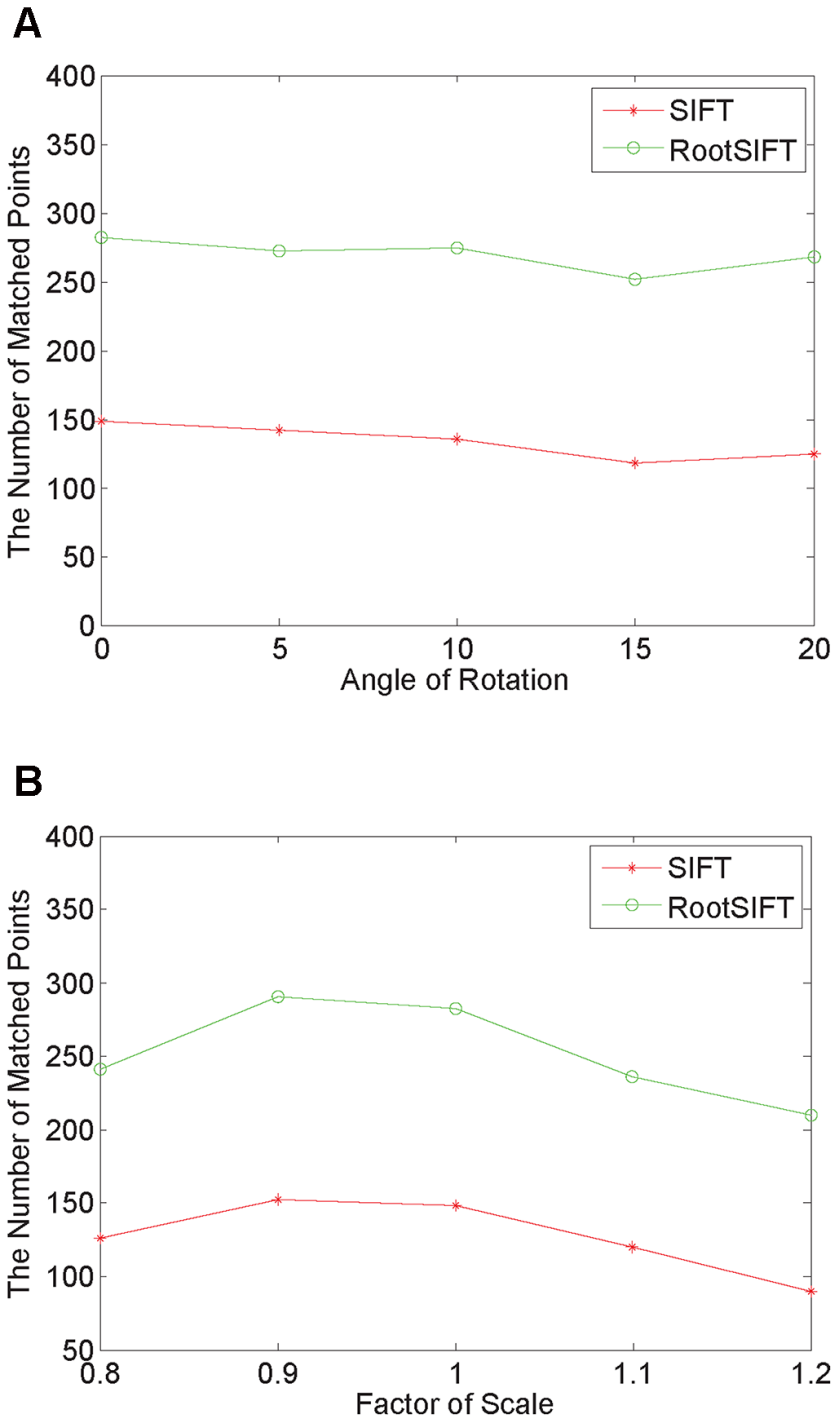
Robustness of SIFT and RootSIFT against rotation and scale transformations. (a) Robustness of SIFT and RootSIFT against rotation transformation; (b) Robustness of SIFT and RootSIFT against scale transformation.

To further compare the differences between SIFT and RootSIFT, we performed different angle rotations and different scale transformations, and then performed feature extraction and matching. The experimental results are shown in Table 1 and Figure 6, which demonstrate that RootSIFT is more robust to changes of scale and rotation than SIFT, and show the efficacy of RootSIFT for the contact-free vein image recognition.

**Table 1:** Robustness of SIFT and RootSIFT against rotation and scale transformations.

	Angle of Rotation					Factor of Scale				
Rotation or scale transformation	0	5	10	15	20	0.8	0.9	1	1.1	1.2
Matching key points in SIFT	148	142	135	118	124	126	152	148	120	90
Matching key points in RootSIFT	282	272	275	252	268	241	291	282	236	210

### 3. Hierarchical mismatching removal algorithm

**Figure 7:**
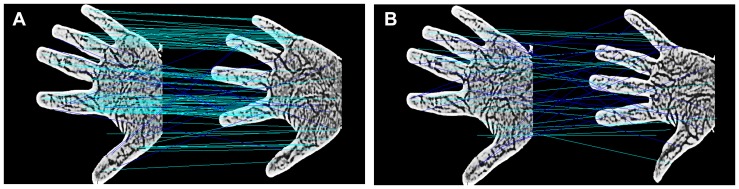
Matching results of the RootSIFT algorithm. (a) Intra-group matching; (b) Inter-group matching.

The above analyses and comparisons demonstrate the improved performance of the RootSIFT algorithm in contact-free palm-vein recognition. However, several mismatching key points for similar textures in different palm-vein images remain. Figure 7 illustrates such a scenario, where the green lines denote correct matching and the blue lines denote mismatching. When compared with intra-group matching as shown in Figure 7(a), there are relatively more mismatching points in the inter-group matching as illustrated in Figure 7(b), which will greatly impact the final recognition performance. To address this problem, some reports [38], [39] have adopted RANSAC [40] to eliminate mismatching. However, RANSAC requires several set parameters and cannot interpret non-rigid transformations, which is often the case in contact-free palm images. Therefore, we propose a new hierarchical mismatching removal algorithm to address this issue including first-layer neighbor-based mismatching removal and second-layer LBP-based mismatching removal.

#### 3.1 Neighbor-based mismatching removal

As shown in Figure 7, the relative distances between the mismatched pairs marked with blue lines are always large; thus, a neighbor-based mismatching removal method is initially adopted to eliminate mismatching points, as described below. (1) Locate the centroids 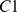 and 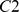 of the two valid palm regions in two images; (2) Compute the relative coordinates 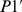 and 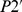 according to location of the matched key points 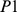 and 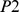 related to 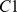 and 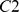, respectively; (3) Compute the Euclidean distance 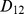 between 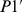 and 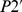; (4) Judge whether 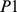 and 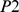 are a mismatching pair according to relationship between 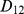 and pre-set threshold, since the Euclidean distance is proportional to the mismatching probability of the two points.

#### 3.2 LBP-based mismatching removal

**Figure 8:**
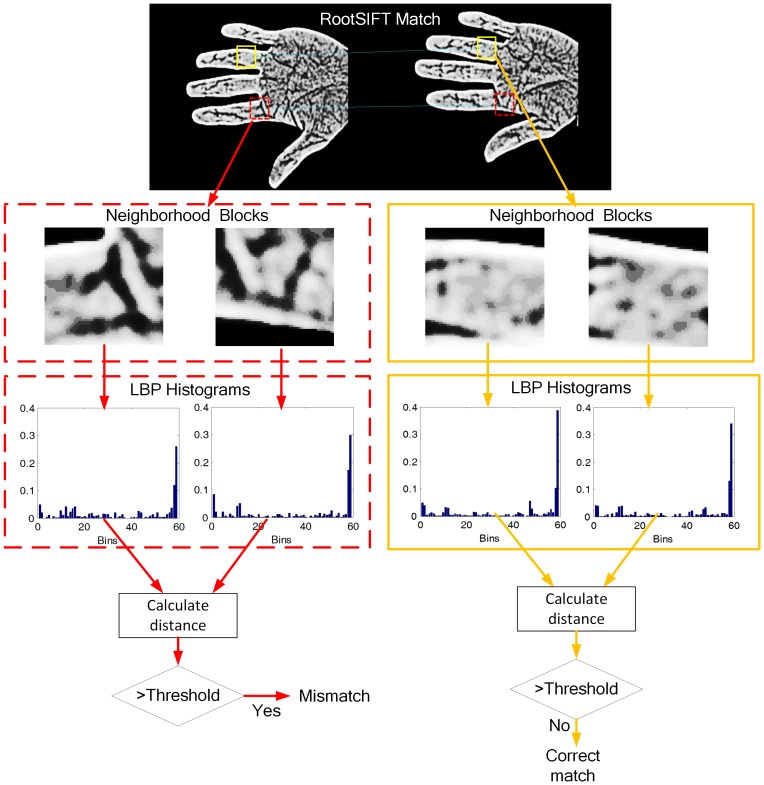
Flow chart of LBP-based mismatching removal.

Following neighbor-based mismatching removal, some mismatching points still remain; thus, the LBP histogram method was adopted to further eliminate mismatching. LBP is a texture descriptor based on gray-level comparisons between neighboring points and a centered point, as originally proposed by Ojala [41] in 1994. The original LBP considers a 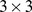 neighborhood of 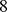 pixels around a center pixel 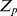, 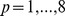, which are binarized with respect to the center pixel; the result is considered a binary number: 
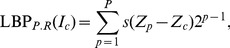
(7)where 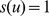 if 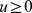, otherwise 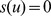. Each binary number is considered a type of micro-pattern. The LBP histogram shows statistical information on these micro-patterns. There are many micro-patterns in the original LBP histogram, but the uniform pattern is used widely for practical applications. The “uniform” LBP is based on the patterns having, at most, two spatial transitions (biswise 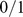 changes). Each pattern in the uniform LBP denotes a particular pattern, such as a line, plane or edge. Because the palm veins are presented as an irregular texture, the LBP was adopted to remove additional mismatching points in our study. The implementation process is described in Figure 8, which includes the following steps. (1) Extract two rectangular regions of fixed size that are centered at two matched points; (2) Compute the two LBP histograms for the two rectangular regions; (3) Compute the distance between the two LBP histograms using the following equation: 
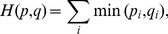
(8)where 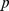 and 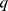 are the LBP histograms of the two rectangular regions to be compared and i represents the 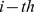 bin; (4) Judge whether the two matched points are mismatched based on pre-set thresholds.

**Figure 9:**
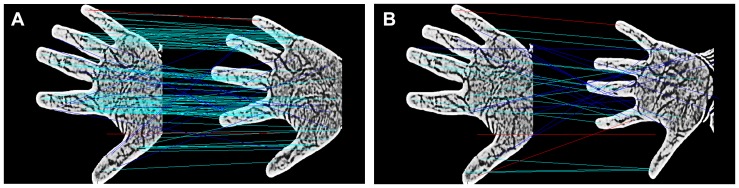
Mismatching removal results. (a) Intra-group matching; (b) Inter-group matching.

The results of the mismatching removal for intra-group and inter-group matching are illustrated in Figure 9(a) and Figure 9(b), respectively, where the red lines denote mismatching removal using on the LBP-based method and the blue lines denote mismatching removal using the neighborhood-based method. As shown in the figures, the proposed hierarchical mismatching removal method eliminates the vast majority of mismatched points. Finally, the number of remaining matching points in the two images is taken as the basis for judging similarity: a higher number of matching points indicates a higher level of similarity between two vein images.

## Results and Discussion

### 1. Ethics Statements

This study had been approved by the Institutional Review Board (IRB) of School of Automation Science and Engineering, South China University of Technology, Guangzhou, Guangdong, China. The CASIA multi-spectral palmprint image database V1.0 [42] was used in this paper to verify the performance of our method. This database is publicly available for palmprint recognition research, and the consent was not needed. The multi-spectral palmprint images and the experimental results are reported in this paper without any commercial purpose. The contact-free palm-vein database was built in our own lab to maintain sufficient variations in posture for performance verification. The 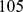 participants gave written consent and all the experiments for this study are without any commercial purpose. All data was anonymized at collection and authors had no access to identifying information other than these images and their reference numbers.

### 2. Databases

**Figure 10:**
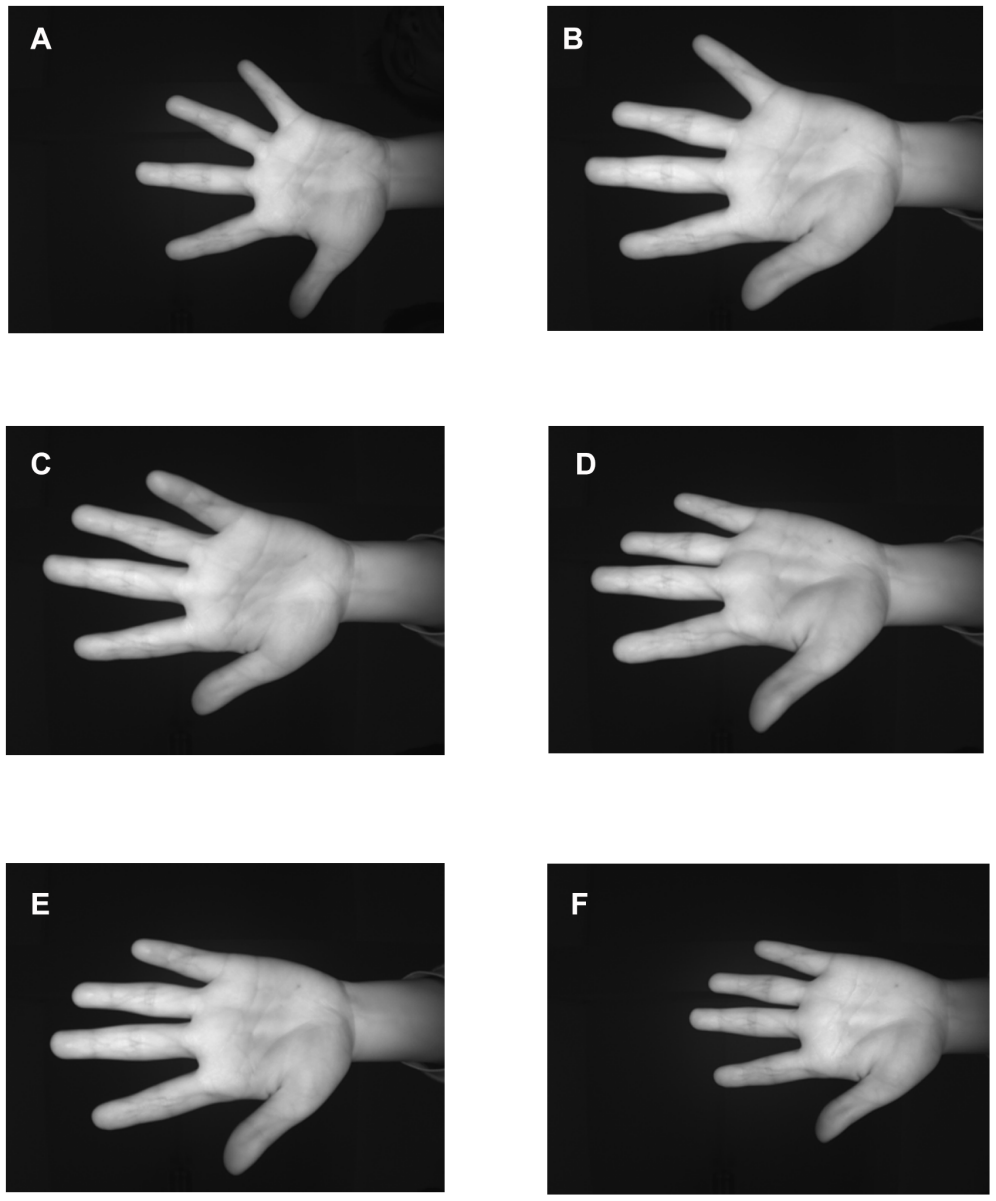
Samples in our database. (a) Far from the camera lens; (b) Close to the camera lens; (c) Tilts to the left; (d) Tilts to the right; (e) Tilts forward; (f) Tilts backward.

To reliably evaluate the effectiveness of the proposed approach, four experiments were conducted using contact-free palm-vein databases. To the best of our knowledge, there is only one public contact-free palm-vein database that is a sub-database of the CASIA Multi-spectral Palmprint Image Database V1.0, so it was adopted as one database to evaluate the proposed approach in our study. All images in the database were acquired in two data acquisition sessions separated by a time interval of more than one month, and at each time, three samples were acquired from each user at six different wavelengths (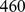 nm, 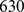 nm, 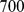 nm, 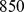 nm, 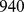 nm and white light). However, in the CASIA database, the majority of hand postures of each individual did not vary as much as would be expected in actual scenarios. To resolve this issue, we built a new contact-free palm-vein database, SCUT palm-vein Image Database,to maintain sufficient variations in posture from each person. In our database, 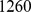 sample images were acquired from the left and right hands of 105 subjects. Six samples from each hand represent six different hand postures: scale variations occur in the first and second samples, as shown in Figure 10(a) and Figure 10(b), tilts to the left or right occur in the third and fourth samples, as shown in Figure 10(c) and Figure 10(d), and tilts forward or backward occur in the fifth and sixth samples, as shown in Figure 10(e) and Figure 10(f).

Several measurements were used to evaluate the performance of our proposed approach, including the false rejection ratio (FRR) and the false acceptance ratio (FAR). The FRR is the probability of falsely declaring an authorized user as an imposter, whereas the FAR is the probability of falsely declaring an imposter as an authorized user. The receiver operating characteristic (ROC) curve between the FRR and the FAR reflects the overall performance of an algorithm. The equal error rate (EER) is the point at which the FRR equals the FAR; the smaller the EER, the better the performance of the algorithm.

### 3. Palm-vein verification

**Figure 11:**
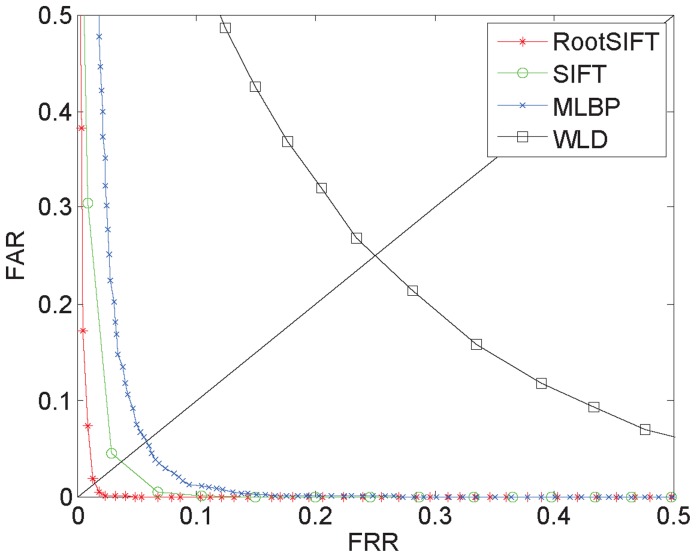
EER curves for different approaches.

The aim of the experiment was to evaluate the performance of our proposed algorithm in recognizing contact-free palm-vein images. Thus, we chose palm-vein images collected at 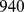 nm NIR illumination for evaluation and compared our approach with SIFT [23], multi-scale LBP [33] and WLD [43]. To ensure consistency, the same hierarchical enhancement method was employed for each case, and the entire palm region was used for the SIFT and RootSIFT recognition, whereas the center rectangular region of the palm was used for MLBP and WLD because these methods are ill-suited for entire palm-region recognition. As shown in Figure 11, palm-vein verification using our proposed approach showed significantly improved performance over the other approaches when using contact-free palm-vein images.

Moreover, we tested our proposed approach using the aforementioned palm-vein database to evaluate its robustness against translation, rotation and scale variance. The results are shown in Table 2. The EER is 0.996% for the CASIA database and 3.112% for our database, which demonstrates that our proposed approach is much more robust to translation, rotation and scale variance than other approaches and that it is suitable for contact-free palm-vein identification.

**Table 2:** Comparative experiments for two different databases.

Databases	Database size	SIFT	MLBP	WLD	Our approach
CASIA palm-vein database	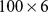	2.207%	4.954%	14.2%	0.996%
Our database	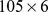	7.056%	9.8%	18.4%	3.112%

Finally, we compare the time consumed in feature extraction and matching of different methods. To make the time consumption proximate to its actual value, 100 randomly selected palm-vein images from CASIA database are utilized for evaluation. We conduct our experiments in the experimental environment of Intel Core (TM) 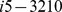 CPU 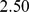 GHZ, and the results are shown in Table 2. As seen from Table 3, the time consumption of our method is a little bit better than that of SIFT, since RootSIFT adopt Hellinger kernel rather than Euclidean distance for calculating the matching distance in feature matching. At the same time, SIFT and our method are more time-consuming than MLBP and WLD, while their more complex feature extraction process bring better recognition rate just as expected. So our method is more competitive as far as comprehensive comparison.

**Table 3:** The time consumption for different methods (s).

Process	SIFT	MLBP	WLD	Our approach
Feature extraction	0.996285	0.115082	0.540108	0.990973
Feature matching	0.289805	0.001874	0.000624	0.268397

### 4. Multi-spectral palmprint image verification

To further verify the robustness of our approach, the sub-database of all palmprint images collected under six different wavelengths in the CASIA multispectral palmprint database was used to perform recognition experiments, and the results are shown in Table 4. As shown in the table, the EERs is relatively lower in the visible spectrum, including 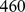 nm, 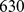 nm and white light, and in the NIR spectrum, including 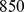 nm and 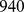 nm. By analyzing the CASIA multi-spectral palmprint images, it is observed that palmprint textures are clearer in images collected in the visible spectrum and palm-vein textures are clearer in images collected in the NIR spectrum. However, both palm-vein and palmprint textures are blurred and less discriminative when the images are collected at 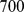 nm, which consequently affect the accuracy of the final recognition. The above analyses suggest that our approach is suitable not only for palm-vein verification but also for palmprint verification.

**Table 4:** Verification results from the CASIA multi-spectral palmprint database.

Wavelength	460 nm	630 nm	700 nm	850 nm	940 nm	WHT
EER	1.728%	1.329%	2.989%	1.825%	0.996%	1.960%

### 5. Effect of different preprocessing methods on palm-vein verification

**Figure 12:**
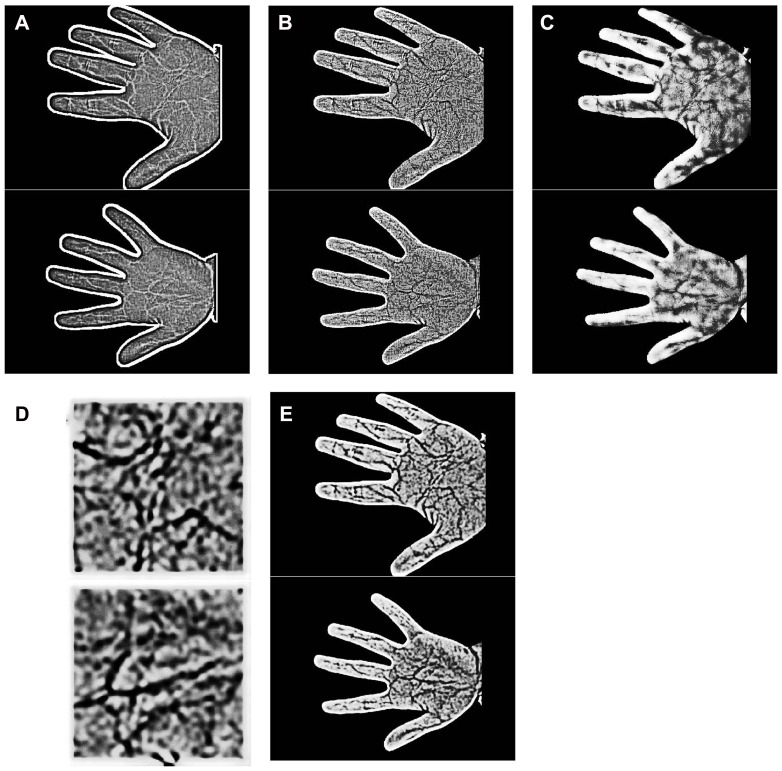
Examples of five preprocessing methods. (a) Weber illumination normalization; (b) Retinex based illumination normalization; (c) Background estimation; (d) Our method on ROI; (e) Our method.

Image preprocessing plays a key role in feature extraction and directly affects feature extraction and matching results, so some literatures have adopt different approaches for image preprocessing before feature extraction, such as, Weber illumination normalization [44], Retinex based illumination normalization [26], [27], and background estimation [7], [35]. In order to illustrate the effectiveness of our proposed preprocessing method, we designed five experiments to compare the effect of different preprocessing procedures on the final recognition results. The first three experiments adopted three aforementioned methods for preprocessing the entire palm region respectively, the fourth experiment extracted the center rectangular region of the palm as the ROI and used our hierarchical enhancement method for preprocessing, the fifth experiment adopted our hierarchical enhancement method for preprocessing the entire palm region. Figure 12 illustrates the final results obtained from the five experiments, which shows that the feature information and vein-texture were markedly better when palm-vein images were preprocessed using our proposed method.

**Figure 13:**
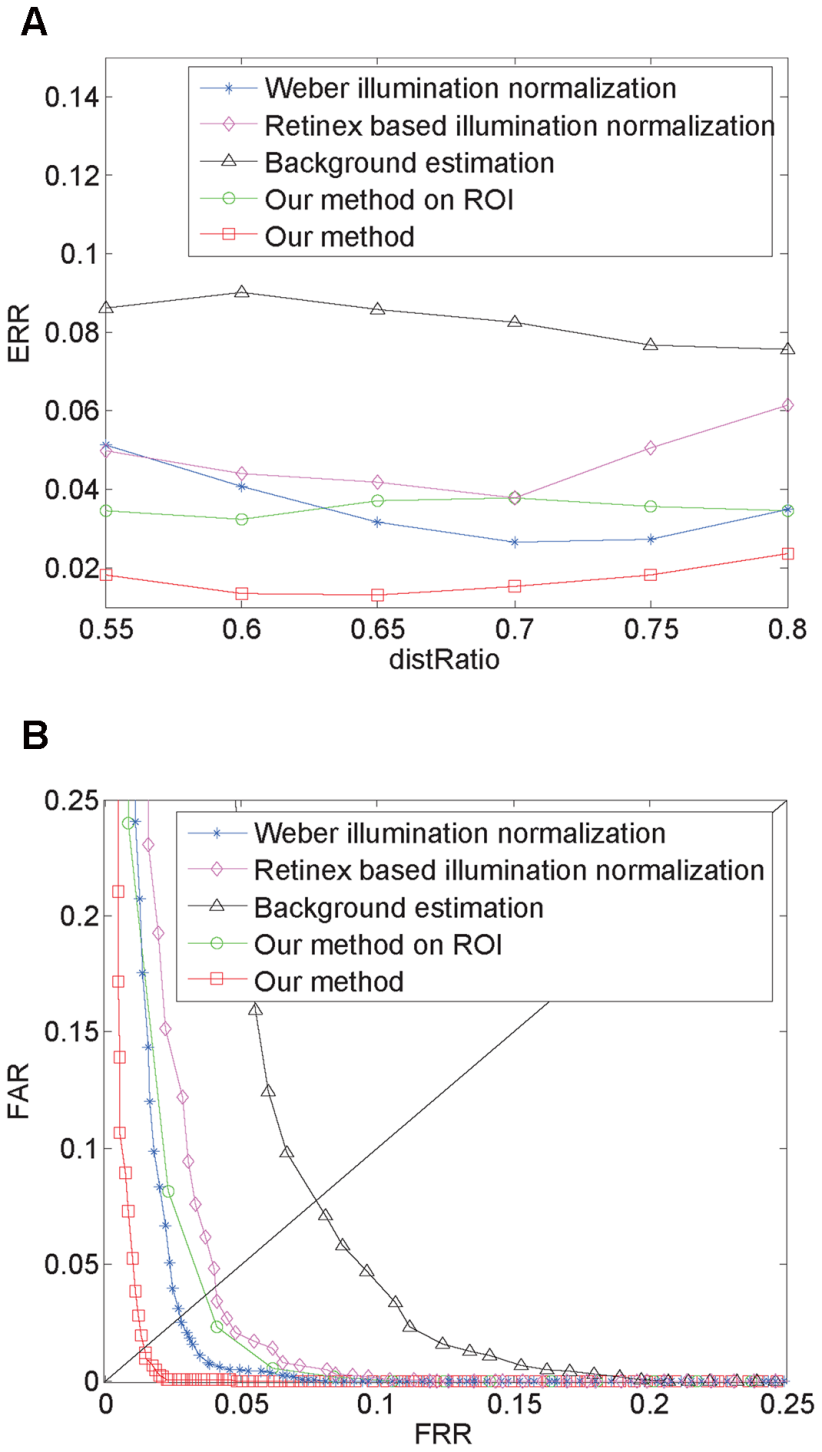
Comparative experiment using five different preprocessing methods. (a) EERs of the five preprocessing approaches for different distRatio values; (b) EER curves for the five preprocessing approaches for an optimal distRatio.

To further verify the effect of these five preprocessing methods on palm-vein recognition and to ensure consistency, the same method (RootSIFT) was adopted to perform feature extraction and matching on the images preprocessed by the five approaches. The EERs of the five preprocessing approaches for different distRatio values, defined by the ratio of the closest and second-closest distance, are illustrated in Figure 13(a), and EER curves for the five preprocessing approaches for an optimal distRatio are illustrated in Figure 13(b). As shown in the figures, our approach performs better than the other approaches because our method acquires greater texture contrast and more feature information.

### 6. Comparison of different methods for mismatching removal

**Figure 14:**
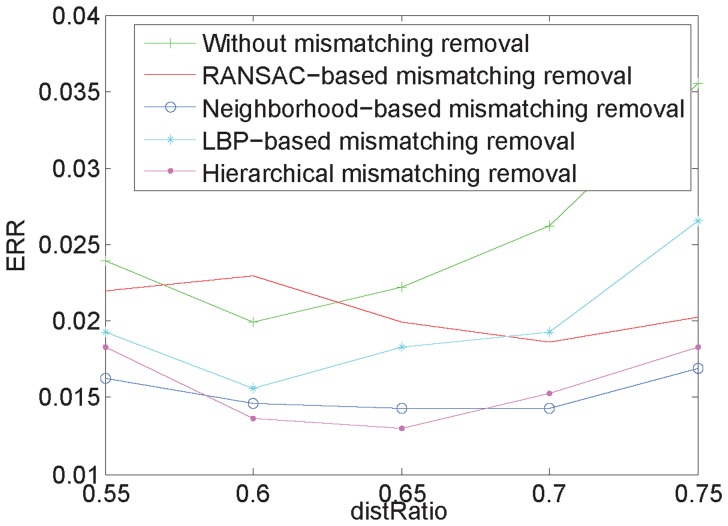
Results from different methods for mismatching removal.

The above analyses clearly show that RootSIFT emphasizes local information on feature points. However, when there are many similar structures in the palm-vein images of different individuals, it is difficult for RootSIFT to distinguish extracted feature points of similar structure from different images, which may lead to mismatches. Thus, we adopted the hierarchical mismatching removal method to increase matching accuracy. To verify our method, five experiments were performed: (1) palm-vein recognition without mismatching removal; (2) palm-vein recognition using RANSAC-based mismatching removal; (3) palm-vein recognition using neighborhood-based mismatching removal; (4) palm-vein recognition using LBP-based mismatching removal; (5) palm-vein recognition using our proposed hierarchical mismatching removal. The results are shown in Figure 14, and they demonstrate the effectiveness of our method.

## Conclusions

To overcome translation, rotation and scale variance in contact-free palm-vein images, we propose a robust palm-vein recognition approach. First, we use the entire palm region for vein recognition, which not only gains more vein information and reduces complexity but also decreases restriction of hand posture during registration and authentication. Second, we propose a novel preprocessing method, DoG-HE, which can effectively enhance vein texture and suppress noise. This step is followed by RootSIFT, a descriptor that is more robust to variations in scale, translation and rotation, which was adopted to perform feature extraction and matching of the preprocessed image. Finally, we propose a hierarchical mismatching removal method to improve the feature matching accuracy. We performed a series of experiments using the CASIA multi-spectral palmprint Image Database, and our proposed approach obtained EERs of 0.996% with 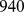 nm palm-vein images and EERs of 1.329% with 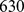 nm palmprint images, which demonstrates that our method is suitable for both palm-vein recognition and palmprint recognition. Moreover, to verify the efficacy and robustness of our proposed method for palm-vein recognition in real-life scenarios, we built a new contact-free palm-vein database containing a large pose variety that included 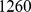 sample images from 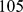 objects, from which we obtained an EER of 3.112%. All of these experiments illustrate the efficacy and robustness of our proposed approach. However, there are also with some limitations. For example, our proposed algorithm is relatively time-consuming when compared with the statistical-based methods such as MLBP and WLD, although the recognition rate of our proposed method surpasses them. Furthermore, since the mobile multi-spectral palmprint recognition is a promising direction, to construct a public mobile multi-spectral palmprint database is urgent for performance evaluation in future work. Therefore, we plan to construct a mobile multi-spectral palmprint database with enough samples and sufficient time interval between data acquisition sessions. On this basis, we will further optimize our algorithm in order to decrease the required time and improve the final accuracy in recognition.
